# Gene Expression Profiling Predicts Survival in Conventional Renal Cell Carcinoma

**DOI:** 10.1371/journal.pmed.0030013

**Published:** 2005-12-06

**Authors:** Hongjuan Zhao, Börje Ljungberg, Kjell Grankvist, Torgny Rasmuson, Robert Tibshirani, James D Brooks

**Affiliations:** 1Department of Urology, Stanford University School of Medicine, Stanford, California, United States of America; **2**Departments of Surgical and Perioperative Sciences, Urology, and Andrology, Medical Biosciences, Clinical Chemistry, and Radiation Sciences, Oncology, Umeȧ University, Umeȧ, Sweden; **3**Department of Health Research and Policy, Stanford University School of Medicine, Stanford, California, United States of America; National Institutes of HealthUnited States of America

## Abstract

**Background:**

Conventional renal cell carcinoma (cRCC) accounts for most of the deaths due to kidney cancer. Tumor stage, grade, and patient performance status are used currently to predict survival after surgery. Our goal was to identify gene expression features, using comprehensive gene expression profiling, that correlate with survival.

**Methods and Findings:**

Gene expression profiles were determined in 177 primary cRCCs using DNA microarrays. Unsupervised hierarchical clustering analysis segregated cRCC into five gene expression subgroups. Expression subgroup was correlated with survival in long-term follow-up and was independent of grade, stage, and performance status. The tumors were then divided evenly into training and test sets that were balanced for grade, stage, performance status, and length of follow-up. A semisupervised learning algorithm (supervised principal components analysis) was applied to identify transcripts whose expression was associated with survival in the training set, and the performance of this gene expression-based survival predictor was assessed using the test set. With this method, we identified 259 genes that accurately predicted disease-specific survival among patients in the independent validation group (*p* < 0.001). In multivariate analysis, the gene expression predictor was a strong predictor of survival independent of tumor stage, grade, and performance status (*p* < 0.001).

**Conclusions:**

cRCC displays molecular heterogeneity and can be separated into gene expression subgroups that correlate with survival after surgery. We have identified a set of 259 genes that predict survival after surgery independent of clinical prognostic factors.

## Abstract 2

Molecular heterogeneity of renal cell carcinomas can be used to distinguish subgroups that correlated with long term survival.

## Introduction

Nearly half of the patients diagnosed with renal cell carcinoma (RCC) succumb to their disease, and RCC accounts for 95,000 deaths per year worldwide [1]. In the United States, approximately 36,160 cases will be diagnosed this year alone, and 12,660 patients will die of their disease [2]. Conventional renal cell carcinoma (cRCC) accounts for approximately 75% of all RCC and accounts for the majority of kidney cancer mortality. Surgery (nephrectomy) can cure 60%–70% of patients with localized disease and prolong survival in patients with metastatic disease, although survival rates after treatment have not changed appreciably in the past 30 y [2,3]. Cytokine therapy, which is reserved for patients with advanced disease, can produce partial responses in 10%–15% of patients and durable remissions in 5% [4].

Tumor stage is the most powerful predictor of outcome in patients with cRCC, although it provides a relatively crude estimate of survival that limits its use in clinical decision making [5]. Several prognostic algorithms have been developed that incorporate tumor stage, grade, and patient performance status, and they predict survival better than stage alone [5–7]. Based on these algorithms, fewer radiographic imaging and blood tests have been proposed for patients predicted to have a low risk of recurrence after surgery, and adjuvant therapy has been suggested for high-risk patients. Unfortunately, many patients fall into intermediate-risk categories, and these algorithms do not predict survival or response to therapy in patients with advanced disease [6].

The limitations of the prognostic algorithms and the varied response to surgery and immunotherapy suggest that cRCCs are molecularly diverse and that capturing relevant molecular features could improve outcome prediction. In support of this idea, several small series used DNA microarray analysis to identify genes whose expression levels correlated with survival in RCC, although the prognostic gene sets did not overlap, and neither study has been validated independently [8–10]. To identify gene expression correlates of survival in cRCC, we used DNA microarrays to explore systematically the molecular variations underlying the biologic and clinical heterogeneity in a set of 177 tumors with associated detailed clinical information, including long-term follow-up.

## Methods

### Samples

Tumors from 177 consecutive patients who underwent radical nephrectomy for cRCC collected between 1985 and 2003 were selected from the fresh-frozen tissue bank in the Department of Urology, Umeȧ University Hospital (Umeȧ, Sweden). Written informed consent was obtained from all patients, and the study was approved by the institutional review board of each participating center. Patients in the study included 102 men and 75 women with cRCC diagnosed on the nephrectomy specimens by pathologists at Umeȧ University Hospital (summarized in Table 1). Mean age of the patients was 65 y (range, 34 to 85 y), and performance status, assessed using World Health Organization criteria, ranged from 0 (65 patients), 1 (64 patients), 2 (37 patients), 3 (ten patients), to 4 (one patient). Pathologic stage grouping of patients in the study, based on preoperative radiographic studies and pathological assessment of the surgical specimens was I (49 patients), II (29 patients), III (40 patients), and IV (59 patients). No patient received neoadjuvant therapy prior to surgery. Adjuvant interferon therapy was given to seven patients and adjuvant hormonal therapy to 12 patients, and all had stage IV disease at the time of surgery. Thirteen patients who recurred after surgery received salvage interferon therapy, nine had resection of metastases, and 19 received hormonal therapy. Patient follow-up status was assessed at least yearly by routine clinical follow-up at Umeȧ University Hospital or by contacting patients directly. Median follow-up of censored patients was 76 mo (range 19 to 224 mo). During the follow-up period, 87 patients died of their disease, 25 died of other causes, nine were alive with disease, and 56 were alive and free of disease.

**Table 1:**
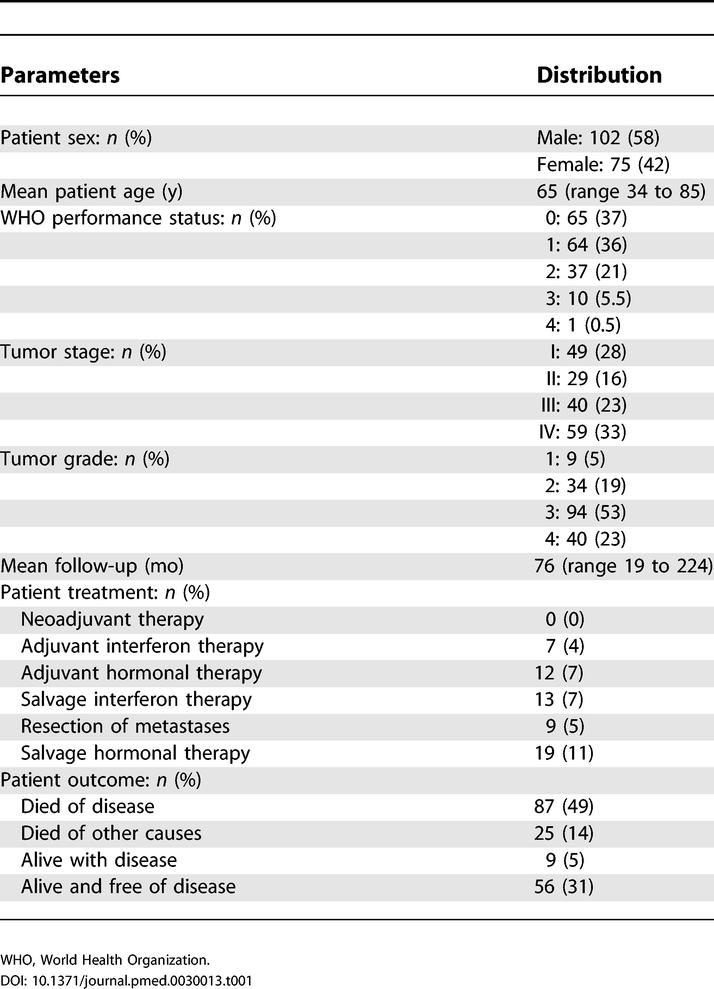
Description of Patients

### Gene Expression Profiling

Total RNA was isolated from the cRCC tissue samples using TRIzol reagent (Invitrogen, Carlsbad, California, United States), according to the manufacturer's recommendations. The integrity of the total RNA was assessed using a 2100 Bioanalyzer (Agilent Technologies, Palo Alto, California, United States). Cy5-labeled total RNA from cRCC samples was mixed with Cy3-labeled Universal Human Reference RNA (Stratagene, La Jolla, California, United States) and hybridized to cDNA microarrays (manufactured by the Stanford Functional Genomics Facility) that contained over 40,000 cDNA clones, representing 27,290 unique UniGene clusters as described previously [11]. Arrays from ten different print runs were used in the study, and all arrays passed a set of quality control criteria defined by GenePix software, including mean of median background less than 500, feature variation less than 0.5, background variation less than 0.5, and features with saturated pixels less than 0.1%. For an explanation of each of these quality control measures, see http://www.moleculardevices.com/pages/software/gn_genepix_pro.html. Microarrays were imaged using an Axon GenePix 4000B scanner (Axon Instruments, Union City, California, United States), and fluorescence ratios of the tumor RNA specimens compared to the reference RNA were determined using GenePix software. Data was entered into Stanford Microarray Database for subsequent analysis [12]. The complete microarray dataset is available at http://smd.stanford.edu/cgi-bin/publication/viewPublication.pl?pub_no=484. The data also have been deposited in National Center for Biotechnology Information's Gene Expression Omnibus (see Accession Numbers section).

### Statistical Analysis

#### Hierarchical clustering analysis

Fluorescence ratios were normalized by mean-centering genes for each microarray, mean-centering each gene across all microarrays, and centering within each of ten microarray print runs (to minimize potential print-run-specific bias). We selected 3,674 genes represented by 5,560 clones on the microarrays whose expression was both well measured and highly variable among samples (a complete list is available at http://smd.stanford.edu/cgi-bin/publication/viewPublication.pl?pub_no=484). We defined well-measured genes as those with a ratio of signal intensity to background noise of more than 1.5 for either the Cy5-labeled cRCC sample or the Cy3-labeled reference sample, in at least 70% of the samples hybridized. Genes with highly variable expression were defined as those whose expression was higher or lower by a factor of at least three than the average expression of all cRCC samples in at least ten cRCC samples. We applied two-way (genes-against-samples) average-linkage hierarchical clustering and used TreeView to visualize the results [13]. We compared the survival times of the five gene expression subgroups using Kaplan-Meier survival analysis and the log-rank test.

#### Supervised principal components analysis

For outcome prediction, we randomly divided samples—that had been prestratified to ensure that a similar proportion of samples in each group were from patients who had died and with similar clinical parameters, including tumor stage grouping, grade, performance status, and length of follow-up—into a separate training set (88 samples) and test set (89 samples) (Table 2). In the training set, we calculated modified univariate Cox proportional-hazard scores for all genes (*n* = 14,814) that were well measured to identify genes whose expression correlated with the duration of survival. (The modification adds a constant to the denominator, as described in [14].) We selected a set of genes whose absolute Cox score statistic exceeded a threshold that was chosen using multiple 2-fold cross-validation. To determine the threshold, the training samples were divided randomly and principal components were derived from half of the samples and then used in a Cox model to predict survival in the other half. We repeated this entire process five times and found that a threshold of ±1.5 yielded the highest average partial log-likelihood ratio statistic. Principal components analysis was then performed on all cases in the training set, using 340 transcripts representing 259 genes whose absolute Cox score equaled or exceeded the threshold. Only the first principal component was associated significantly with survival. For patients in the test set, a continuous risk score (that is, the supervised principal components [SPC] risk score) was calculated for each patient, based on transcript levels across the 340 transcripts and the weights assigned to each transcript derived from SPC analysis of the training set. Multivariate proportional-hazards analysis was performed on the test set with the SPC risk score as a continuous variable, along with stage grouping, grade, performance status, and gene expression subgroup derived from hierarchical clustering analysis.

**Table 2:**
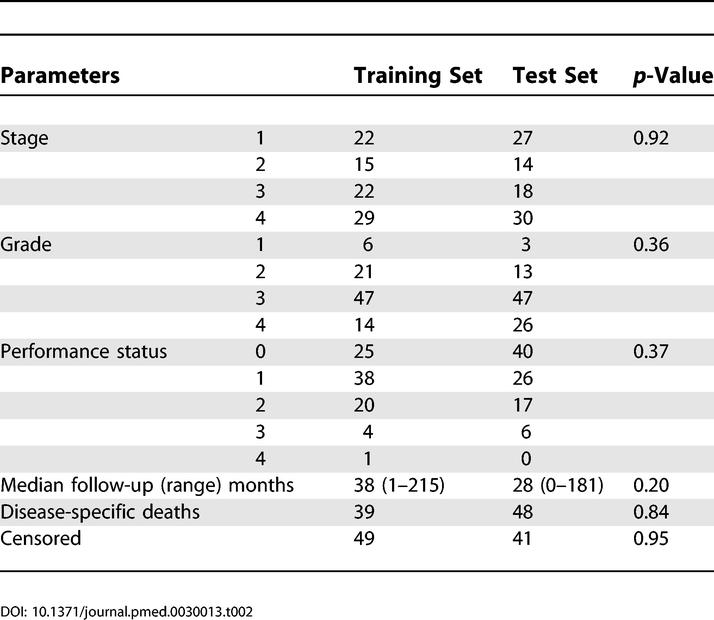
Patient Distribution between Training and Test Set

To evaluate the gene set as a categorical predictor of survival, we divided the training set into tertiles based on the SPC risk scores. The test set then was divided into three groups based on the tertiles of the SPC risk scores of the training set. We compared the survival times of the three subgroups in the training and test sets using Kaplan-Meier survival analysis and the log-rank test.

SPC analysis and multivariate proportional-hazards analysis were performed with the use of the R software package (available at www.r-project.org) and the superpc R package (available at http://www-stat.stanford.edu/~tibs/superpc). Kaplan-Meier survival analysis was performed with WinStat software (R. Fitch Software, Staufen, Germany).

## Results

### Gene Expression Profiles of cRCCs

**Figure 1:**
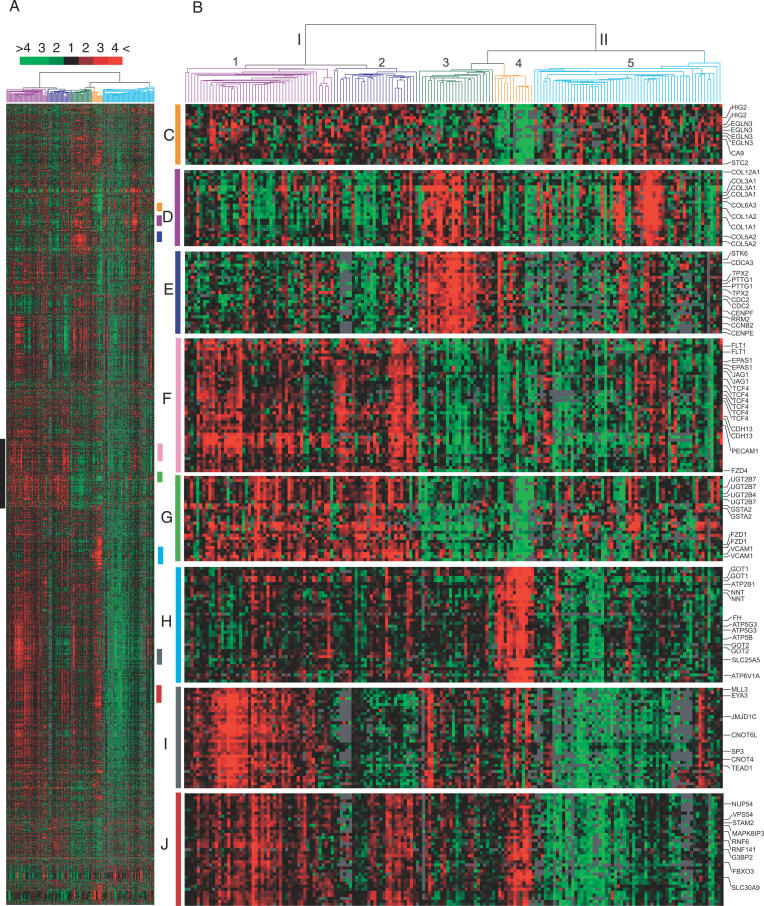
Unsupervised Hierarchical Clustering Analysis of 177 cRCCs (A) Overview of the gene expression patterns of 3,674 genes whose expression varied more than 3-fold in at least ten samples across the 177 samples. Each row represents a single gene, and each column an experimental sample. Colored bars identify the locations of the inserts in (C–J). The degree of color saturation corresponds with the ratio of gene expression shown at the top of the image. (B) Dendrogram representing similarities in the expression patterns between experimental samples. Samples were separated into two main groups and five subgroups (one in purple, two in blue, three in dark green, four in orange, and five in light blue) by the clustering algorithm. (C) Hypoxia-induced gene cluster. (D) Collagen gene cluster. (E) Proliferation gene cluster. (F–I) Genes distinguishing the two main groups (subgroups 1 and 2 from subgroups 3, 4, and 5). (H) Energy generation gene cluster. (J) Genes downregulated uniquely in subgroup 5.

Hierarchical clustering analysis of the 177 patient samples described in Table 1 was performed using 5,560 clones representing 3,674 unique genes whose expression varied more than 3-fold from the mean expression ratio (specimen RNA/reference RNA) in at least ten samples (Figure 1). A detailed view of the sample cluster dentodrogram is displayed in Figure S1. Tumors were partitioned into two main groups and five subgroups based on the differential expression of these 3,674 genes (Figure 1A). The grouping of the tumors in the dendrogram did not appear to be an artifact of the genes used to generate the cluster because varying data-filtering criteria (and the number of genes used in the hierarchical clustering analysis) resulted in a similar pattern of specimen clustering. A large and diverse set of genes distinguished the two main groups of tumors, all of which showed relatively high expression in tumors in subgroups 1 and 2 compared to subgroups 3, 4, and 5 (black bar in Figure 1A). These genes are involved in a variety of biological processes, including angiogenesis *(FLT1, EPAS1,* and *JAG1),* the Wnt signaling pathway *(FZD1, FZD4,* and *TCF4),* cell adhesion *(CDH13, PECAM1,* and *VCAM1),* and cellular metabolism *(UGT2B7, UGT2B4,* and *GSTA2).*

Each of the five subgroups of tumors displayed distinct gene expression patterns. Examples of clusters whose expression patterns distinguished between the subgroups are shown in Figure 1B. Expression patterns in subgroups 1 and 2 were largely similar, although they differed in a set of genes involved in diverse biological processes, including the transcriptional regulators *MLL3, EYA3, JMJD1C, CNOT4, CNOT6L, SP3,* and *TEAD1* (Figure 1I). Compared to the other cRCCs, those in subgroup 4 showed lower expression of many hypoxia-regulated genes (e.g., *HIG2, EGLN3, CA9,* and *STC2*) (Figure 1C). Conventional RCCs commonly harbor *VHL* gene mutations that result in increased expression of hypoxia-regulated genes, suggesting that subgroup 4 cancers either lack inactivating *VHL* mutations or downregulate hypoxia signaling pathways [15,16]. Subgroup 4 tumors also showed increased expression of many genes that characterize chromophobe carcinomas and oncocytomas, including *KIT* and the mitochondrial genes *NNT, FH, GOT1, GOT2, SLC25A5, ATP2B1, ATP5G3, ATP5B,* and *ATP6V1A* (Figure 1H). We have previously observed similar expression patterns in a subset of cRCCs that have granular cytoplasm [17], and a review of the pathological specimens revealed that 11 of 13 tumors in subgroup 4 were conventional carcinomas with granular cytoplasm. Subgroup 3 showed much higher expression of proliferation-associated genes compared to other tumors *(CDCA3, CDC2, CENPE, CENPF, RRM2,* and *CCNB2),* suggesting a higher proliferative activity in these tumors [18,19] (Figure 1E). Interestingly, there was little correlation between expression levels of the hypoxia-regulated genes and proliferation-associated genes (Pearson's correlation coefficient of 0.22), suggesting that higher proliferation activity does not render cRCCs hypoxic and highlighting that expression of hypoxia-regulated genes is an intrinsic feature of most cRCCs. Subgroup 3 tumors (and some subgroup 5 tumors) also showed high expression of several collagen genes *(COL12A1, COL3A1, COL6A1, COL1A1, and COL5A2),* and high expression of collagen genes has been associated with poor prognosis in several tumor types [20] (Figure 1D). Subgroup 5 uniquely displayed decreased expression of a large set of genes that prominently included several membrane transporters *(NUP54, VPS54, STAM2, MAPK8IP3, G3BP2,* and *SLC30A9)* (Figure 1J). The distinct gene expression profiles of each of the subgroups suggest that cRCCs are molecularly heterogeneous despite their similar histological appearance.

### Gene Expression Subgroups of cRCC Differ in Their Clinical Behavior

**Figure 2:**
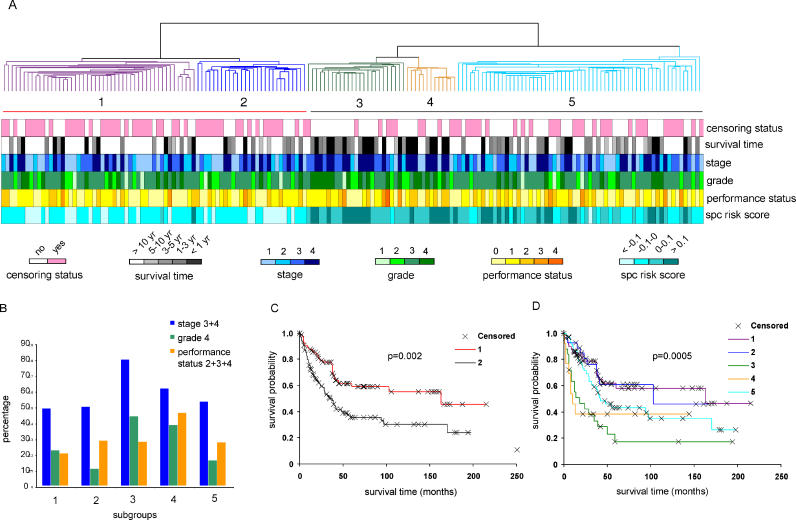
Relationship of Gene Expression Subgroups to Clinical Parameters and SPC Risk Score in 177 cRCCs (A) Dendrogram from the hierarchical cluster, with the clinical information for each of the samples. Subgroups are color-coded as in Figure 1. Color shade corresponds to the ranges of each of the clinical parameters displayed. Expected survival times for the censored observations were estimated from the Kaplan-Meier curve for all patients. (B) Distribution of stage, grade, and patient performance status among five subgroups. (C) Kaplan-Meier estimates of disease-specific survival in the two main gene expression groups of patients (subgroups 1 and 2 shown by the red bar below the dendrogram, compared to subgroups 3, 4, and 5 designated by the green bar). (D) Kaplan-Meier estimates of disease-specific survival in the five subgroups of patients. The X symbols in (C) and (D) denote censored data.

The gene expression subgroups did not simply reflect differences in stage, grade, or performance status since none of these clinical parameters was significantly associated with tumor subtype (*p* > 0.5 by the chi-square test) (Figure 2A and 2B). The two main groups of cRCC defined by unsupervised hierarchical clustering analysis showed a small but significant difference in survival (subgroups 1 and 2 compared to subgroups 3, 4, and 5, *p* = 0.002 by the log-rank test) (Figure 2C). The five expression subgroups better defined classes of tumors that differed in their long-term survival. Kaplan-Meier analysis showed that patients with tumors in subgroup 3 had the worst outcome and those in subgroups 1 and 2 the best compared to other subgroups (*p* < 0.001 by the log-rank test) (Figure 2D). Furthermore, multivariate analysis showed that the expression subgroup was a powerful predictor of survival and was independent of grade, stage grouping, and performance status (*p* = 0.005, by the Cox model likelihood ratio test). Therefore, gene expression profiles separate cRCC into five subgroups that differ prognostically and that reflect differences in the behavior of the tumors not captured by stage, grade, and performance status.

### Gene Expression-Based Survival Predictor

Having identified gene expression signatures in tumors at the time of diagnosis that predict outcome by unsupervised methods (i.e., based purely on gene expression signatures intrinsic to the tumors), we attempted to define a gene expression-based survival predictor by correlating survival time with the gene expression signatures. We have found that supervised analyses that correlate gene expression with disease recurrence or survival (as binary outcome variables) or duration of survival are overly simplistic models of these complex datasets and in general not very accurate at predicting clinical outcomes. We have instead used “semisupervised” learning approaches to identify gene sets associated with survival in adult acute myeloid leukemia and diffuse B-cell lymphoma and have shown that they better identify gene expression signatures that are correlated with outcome compared to unsupervised and supervised methods of data analysis [11,21]. Whereas unsupervised methods assign tumors to a class based solely on gene expression, and supervised approaches use clinical outcome data to select genes associated with prognosis, semisupervised methods combine the advantages of both.

**Figure 3:**
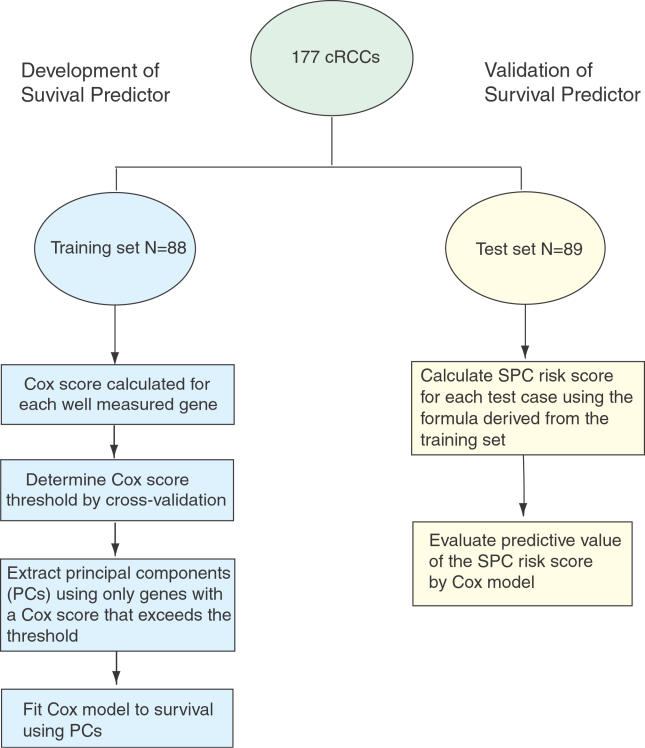
Overview of the Strategy Used for the Development and Validation of a Prognostic Gene List

To identify genes highly correlated with survival in cRCC, we used SPC analysis, a novel semisupervised approach we have developed recently [14,22]. Patient samples were divided randomly into a training set of 88 cases and a test set of 89 cases that were balanced for stage grouping, grade, patient performance status, and length of follow-up (Table 2). In the training set, a modified Cox score was calculated for all well-measured genes, and genes whose Cox score exceeded a threshold that best predicted survival were used to carry out unsupervised principal components analysis (Figure 3). To determine the Cox threshold, we split the training set, performed principal components analysis in one half of the samples and used the model to predict survival in the other half. By varying the threshold of Cox scores and using 2-fold cross-validation, we found that a threshold of ±1.5 (averaged over five separate repeats of this procedure) best predicted survival (i.e., yielded the highest average partial log-likelihood ratio statistic).

**Figure 4:**
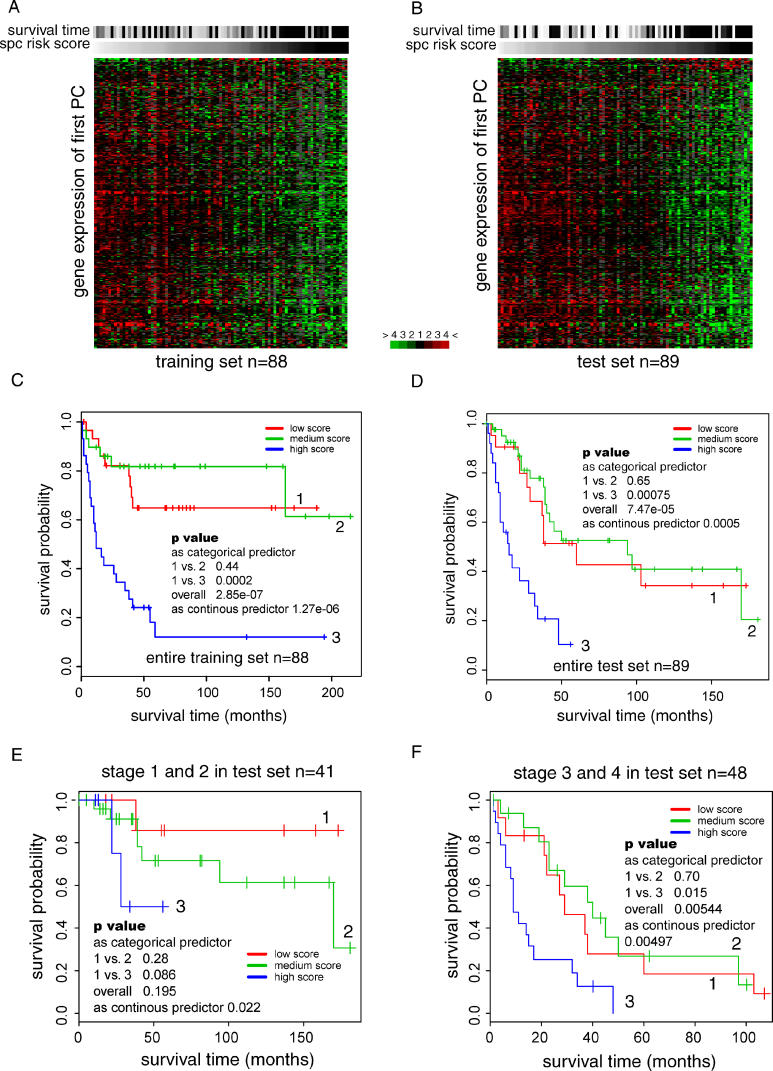
Outcome Prediction Using the SPC Risk Score (A) Overview of the gene expression patterns of the 259 prognostic genes in the training set, with their SPC risk scores arranged in ascending order and the survival time in descending order. Each row represents a single gene, and each column a patient sample. The degree of color saturation corresponds to the ratio of gene expression in each sample compared to the mean expression across all samples. (B) Gene expression profiles of the 259 prognostic genes in the test set. (C) Kaplan-Meier estimates of disease-specific survival in low-, intermediate-, and high-risk groups of patients in the training set defined by the tertiles of SPC risk scores. (D) Kaplan-Meier estimates of disease-specific survival of low, intermediate and high-risk groups of patients in the test set defined based on the tertiles of the SPC risk scores of the training set. (E) Kaplan-Meier estimates of disease-specific survival in stage group I and II patients in the test set. (F) Kaplan-Meier estimates of disease-specific survival in stage III and IV patients in the test set.

There were 340 transcripts (representing 259 genes whose Cox score equaled or exceeded this threshold), and they were used to perform principal components analysis on the entire training set. (For a full list of transcripts with unigene cluster ID, locus link ID, gene symbol, and gene ontology annotations, see Table S1.) As can be seen in Figure 4, only the first principal component was strongly correlated with survival. In 247 genes, high expression levels were associated with prolonged survival (Figure 4A), and in only 12 genes was high expression associated with shorter survival, including *BAG2, DCBLD2, EDG2, GNAS, IGLC2, NCF1, NME2, PFN2, PRPS2, REG4, SLC7A5,* and *TFAP2C.* There did not appear to be enrichment of gene ontology annotations in this prognostic gene set. However, some expression features suggested biological processes that underlie differences in tumor behavior. For instance, three genes involved in adhesion and diapedesis of lymphocytes, CD34, PECAM1, and VCAM1, show higher expression in tumors with good prognosis, and a lymphocytic-mediated immune response can alter the clinical course of cRCCs [4]. Furthermore, high expression of VCAM1 has been shown previously to predict survival in cRCC patients with metastatic disease [9]. Elevated expression of FZD2 and TCF4, members of the Wnt signaling pathway, also correlated with longer survival.

For each case, SPC analysis computed a risk score (SPC risk score) that represents the sum of the weighted expression levels for each of the 340 prognostic transcripts. Not surprisingly, the SPC risk score was highly correlated with survival in the training set (*p* < 0.001 by the log-rank test). To validate the SPC predictor, we computed risk scores for each of the 89 cases in the test set, using the model developed in the training set, and tested whether these scores were correlated with survival (Figure 4B). When the SPC risk score was used as a continuous variable, it was a strong predictor of survival in the independent test set (*p* < 0.001 by the log-rank test). Fewer genes from the SPC set also could predict outcome since genes were identified based on their correlation with survival. For instance, the top four genes in the SPC predictor could be used to predict survival in the test set at *p* = 0.02. Moreover, multivariate analysis showed that the SPC risk score provided powerful prognostication independent of stage, grade, and performance status (*p* < 0.001, by the Cox model likelihood ratio test) (Table 3). Further investigation showed that the log-relative risk was fairly linear in the SPC score. When cases in the test set were split into localized (stages I and II) and advanced disease (stages III and IV), SPC risk score as a continuous variable continued to be highly correlated with survival and was independent of grade and performance status (*p* = 0.011, and *p* = 0.045, respectively, by the Cox model likelihood ratio test).

**Table 3:**
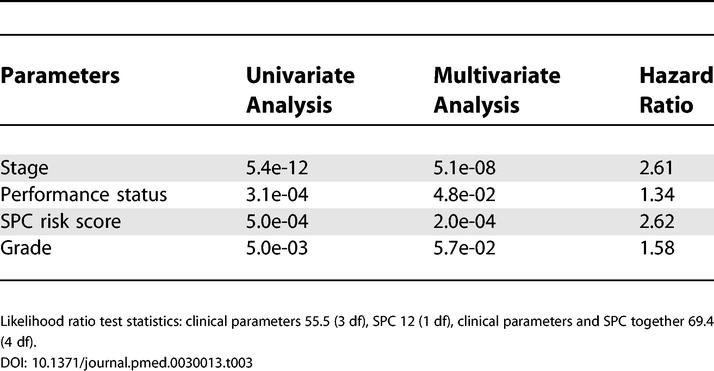
Prognostic Significance of SPC Risk Score Compared to Clinical Features (*p*-Values) by the Log-Rank Test Using the Test Set Samples

To illustrate the performance of the SPC risk score in predicting survival, we divided the training and test sets into tertiles based on the SPC risk scores of the training set. In both the training and test sets, Kaplan-Meier analysis showed that the group with the highest SPC risk score had significantly worse survival compared to the other two groups (Figure 4C and 4D). When used as a categorical predictor, the SPC risk score again predicted survival independent of grade, stage, and performance status in all tumors in the test set and in the high-stage tumors (stage groups III and IV; see Figure 4F), although not in the low-stage tumors (stage groups I and II; see Figure 4E), likely due to low numbers of high-risk cases in the test set. It should be emphasized, however, that the SPC risk score is continuous, and survival is directly correlated with the SPC risk score. Although cases can be assigned to risk categories based on the SPC risk score, outcome is better predicted when the SPC risk score is used continuously, rather than categorically (Figure 4C–4F).

### Relationship of SPC Risk Score to the Gene Expression Subgroups

Most of the 259 prognostic genes comprising the SPC risk score were found in clusters that distinguished between the two major groups of cRCC that were defined by unsupervised hierarchical clustering analysis (i.e., between subgroups 1 and 2 and subgroups 3, 4, and 5) (see Figures 1A [black bar] and 2C). Despite this overlap, the SPC risk score predicted outcome independent of tumor subgroup in the test set (*p* = 0.0013, by the Cox model likelihood ratio test), even though the tumor subgroup had been assigned in the original hierarchical cluster of all 177 tumors (comprising both the training and test sets) and was based on differences in expression over 3,674 genes. Tumor subgroup, on the other hand, did not predict survival independent of the SPC risk score (*p* = 0.12 by the Cox model likelihood ratio test).

## Discussion

We have identified gene expression patterns that correlate with survival after nephrectomy in cRCC. We used unsupervised hierarchical clustering analysis to identify five distinct subgroups that differed in their expression patterns over 3,674 genes. These subgroups were correlated with survival time after surgery that was independent of tumor stage, grade, and patient performance status. The consistency of gene expression within the subgroups, regardless of tumor stage, suggests that distinct molecular genetic changes present at early stages of tumor development determine the fate of the cancer and can be used to predict clinical outcome. The identification of these five new subgroups supports the use of gene expression profiling for prognostication in cRCC and highlights the value of unsupervised analytic methods to provide insights into the clinical and biological heterogeneity of cRCC.

We used a novel, semisupervised analytic strategy to identify 259 genes that better predicted survival than the gene expression subgroups, and we have validated this prognostic gene set on an independent group of patients. We used SPC analysis to compute a continuous risk score that predicted survival in the test set independent of stage, grade, performance status, and gene expression subgroup. Combining the SPC risk score with tumor grade, stage, and patient performance status may help identify patients with cRCC who have a high probability of being cured of their disease and need less intensive follow-up testing after surgery, or high-risk individuals who might be referred for adjuvant treatments even though their disease is clinically occult. The SPC method employed in this paper could be used generally in microarray studies to correlate gene expression with survival time. Other approaches, such as the model-based mixture proposal of Jones et al., could also be tried [23].

An interesting feature of the SPC prognostic gene set is that 95% of the genes show relatively high levels of expression in patients with good outcome and low expression in those with poor outcome. Notably, this pattern of gene expression was observed in a set of 51 prognostic genes identified in 29 cRCCs by Takahashi et al., and 15 of these genes were found in the SPC gene set [8]. Unfortunately, we were not able to evaluate the usefulness of the SPC gene set in predicting survival in their patients because their dataset is not publicly available. Another study by Vasselli and coworkers identified 45 genes associated with survival based on the Cox proportional-hazards score, using 58 stage IV tumors from patients with good performance status [9]. Those genes share minimal overlap (one out of 45) with the SPC gene set, possibly because this study included a highly selected group of patients with tumors of conventional and nonconventional histology. We and others have reported striking differences in transcript profiles of renal cancers of different histologies, and these differences could significantly influence the gene identified that correlate with prognosis [17,24].

The 259 prognostic genes do not show enrichment for any single biological pathway and are not localized to a single region of the genome. The diversity of the pathways represented in the SPC gene set argues that expression of different functional groups of genes contributes to cRCC growth, metastasis, and lethality.

Several molecules have been shown to correlate with prognosis in cRCC; however, most of them were not selected by SPC analysis as strong predictors of survival in our dataset [25,26]. While some of these molecules might have important biological roles in cRCC progression, they could be excluded from the SPC gene list because they are relatively weak predictors of survival. For instance, some transcripts, like *ADFP* (a hypoxia-induced gene) did not provide a strong enough correlation with survival to make the SPC gene set. However, in our dataset, *ADFP* was correlated with a number of the SPC genes in a cluster that defined main tumor groups I and II (see Figure 1) by unsupervised clustering analysis. When the 177 cases were separated into two groups based on their median expression level of *ADFP,* they displayed significantly different cancer-specific survival (*p* = 0.03). *Carbonic anhydrase 9,* another potential prognostic marker for advanced RCC showed uniformly low expression in subgroup 4, which has the worst survival rate, although its expression did not correlate with survival in the whole dataset. Therefore, predictions of outcome that are based on single genes will be less robust that that for multiple genes, such as those of the SPC predictor.

Gene expression profiling can improve outcome prediction in patients with cRCC beyond that provided by stage, grade, and patient performance status. Application of the SPC risk score in the clinical setting will depend on independent confirmation of our findings and could occur through custom DNA microarrays or quantitative reverse-transcriptase polymerase chain reaction assays [27–29]. Since as few as four genes in the SPC gene set can estimate prognosis, it should be possible to develop clinically useful predictors of survival based on these technologies. Regardless, our study demonstrates the molecular heterogeneity of cRCC and opens opportunities for improved biological understanding of the molecular subgroups of the disease and their response to therapy.

## Supporting Information

Figure S1 Dendrogram Representing the Similarities in the Gene Expression Patterns between Experimental Samples (451 KB PDF).

Table S1 The 259 Genes Predicting Survival Identified using SPC (65 KB XLS).

### Accession Numbers

The complete microarray dataset has been deposited in National Center for Biotechnology's Gene Expression Omnibus (http://www.ncbi.nlm.nih.gov/geo/) and is accessible through accession number GSE3538.

**Patient Summary**

**Background**

The kidneys filter the blood and eliminate waste in the urine through a complex system of filtration tubules. All of the blood in the body passes through the kidneys approximately 20 times an hour. Conventional renal cell carcinoma (cRCC) is the most common type of kidney cancer and arises from the cells that line the filtration tubules of the kidney. Nearly half of the people who get RCC die from the disease. Gene expression profiling, a laboratory technique, offers promise for guiding the diagnosis and treatment of cancers.

**Why Was This Study Done?**

The current method for estimating survival using clinical markers (such as tumor stage and grade) has limitations. As has been found for other cancer types, the hope is that gene expression profiling could identify molecular markers that could be used for more accurate diagnosis, prognosis, and possibly serve as drug targets for effective therapies. Several small gene expression studies of cRCC done so far have each identified prognostic gene sets, but these genes did not overlap, and studies have not been validated independently. This larger study looked systematically for variations in gene expression that were correlated with the clinical heterogeneity of cRCCs.

**What Did the Researchers Do and Find?**

The researchers studied a set of 177 tumors from patients for whom they had detailed clinical information, including data on long-term survival. They found a set of 259 genes whose activity in the tumor correlated with long-term survival independent of the standard clinical predictors. Most of the genes showed high levels of expression in patients with good outcome and low expression in those with poor outcome. They then used this information to show that they could accurately predict survival in an independent group of patients.

**What Do These Findings Mean?**

The researchers identified a set of genes whose activity predicted survival after surgery independent of clinical prognostic factors. This suggests that expression profiles could help to distinguish between more aggressive and less aggressive types of cRCCs. If confirmed by other studies, such expression profiles could be used with information on tumor grade, stage, and patient performance to help identify patients with cRCC who have a high probability of being cured and need less intensive treatment and follow-up testing after surgery and others whose cancers should be treated more aggressively.

**Where Can I Get More Information Online?**

The following Web sites have information on kidney cancer.

Cancer Research UK:

http://www.cancerresearchuk.org/

MedlinePlus:

http://www.nlm.nih.gov/medlineplus/ency/article/000516.htm

CancerBACUP:

http://www.cancerbacup.org.uk/Cancertype/Kidney

US National Cancer Institute:

http://www.cancer.gov/cancertopics/types/kidney

CancerLinks:

http://www.cancerlinks.com/kidney.html

Kidney Cancer Association:

http://www.kidneycancerassociation.org/

## Abbreviations

cRCC: conventional renal cell carcinoma
RCC: renal cell carcinoma
SPC: supervised principal components
