# Supplementary material for: Gene Expression Profiling Predicts Survival in Conventional Renal Cell Carcinoma
Source: PLoS Med. 2005 Dec 6;3(1):e13. doi: 10.1371/journal.pmed.0030013 (PMC1298943; doi:10.1371/journal.pmed.0030013)

Supplemental Figure

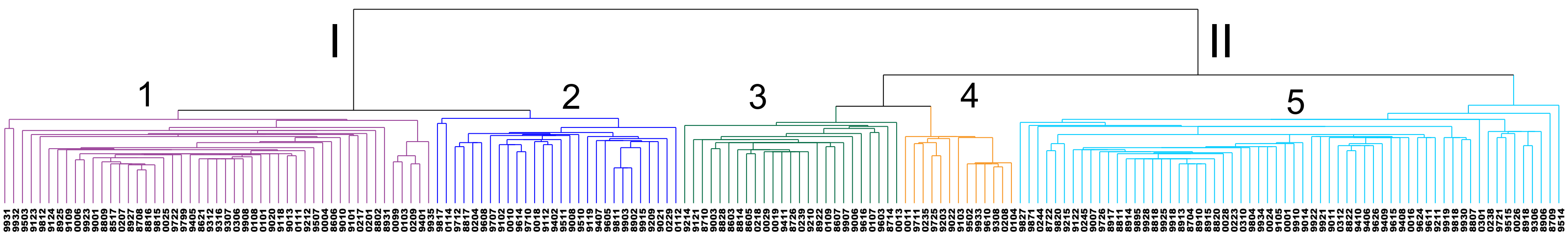

Supplement: Supplementary file 1 [file pmed.0030013.sg001.pdf]
